# Annual Homicide Rate as a Proxy for Overall Gun-Related Violent Crime: A Retrospective Study

**DOI:** 10.7759/cureus.86544

**Published:** 2025-06-22

**Authors:** Richard H Lewis, Louis J Magnotti, Nathan Manley, Greggory R Davis, Benjamin Martinez, William Hoover, Tomas Jacome

**Affiliations:** 1 Trauma Surgery, Our Lady of the Lake Regional Medical Center, Baton Rouge, USA; 2 Trauma Surgery, The University of Arizona, Tucson, USA; 3 Trauma Surgery, Banner Health, Casper, USA; 4 Emergency Medicine, Louisiana State University Health Sciences Center, Baton Rouge, USA; 5 Office of Research and Grant Administration, Our Lady of the Lake Regional Medical Center, Baton Rouge, USA; 6 Surgery, Louisiana State University Health Sciences Center, New Orleans, USA

**Keywords:** community perception, firearm-related death, gunshot injuries, murder, non-fatal gunshot wounds, police reports

## Abstract

Introduction

The annual reporting of homicides often captures the nation’s attention, with the unspoken assumption that this serves as a proxy for overall gun-related violent crime. The purpose of this study is to evaluate whether the overall trend in homicide rates accurately reflects the overall trend in gun-related violent crime over time.

Methods

Using police crime incident data from a large urban city in the southern United States, the total number of gun-related violent crimes, gunshot victims treated at the trauma center, and homicides per year from 2014 to 2020 were recorded. Rates of gunshot victims, gun-related violent crimes, and homicides per 100,000 population were stratified by year and compared over time using simple linear regression. Analysis of covariance (ANCOVA) was then used to compare the rate of increase of homicides to gun-related violent crimes.

Results

There were 4,928 gun-related violent crimes and 567 homicides over the study period. Linear regression analysis identified a significant increase in gun-related crime rate (201.92 to 447.3 incidents per 100,000 population, p = 0.01) and homicide rate (25.90 to 50.24 incidents per 100,000 population, p = 0.028) from 2014 to 2020. The rate of gunshot victims treated at the trauma center appeared to increase during the study period, although the increase was not statistically significant (57.94 to 125.6 incidents per 100,000 population, p = 0.0624). ANCOVA revealed that gun-related violent crimes increased at a greater rate compared to homicides, with respective slopes of 34.01 (95% CI: 20.49 to 47.54) and 3.36 (95% CI: -15.77 to 36.03). The interaction between year and crime type was statistically significant (p = 0.005), indicating different rates of increase.

Conclusion

Annual homicide rates should be interpreted with caution, as they may not accurately reflect the true extent of gun violence in communities. Broadening our understanding represents the first step in preventing continued increases in this major public health problem.

## Introduction

Gun violence remains a significant public health problem in the United States. Despite efforts to address this epidemic of gun violence, gun-related deaths have steadily increased over the past several decades. For example, there were 48,204 gun-related deaths in 2022, compared to 31,672 in 2010 and 28,663 in 2000 [1]. During these years, gun-related homicides accounted for 41% (n = 19,651), 35% (n = 11,078), and 38% (n = 10,801) of all gun-related mortality, respectively [1]. For context, firearms accounted for even more deaths than motor vehicle accidents in 2022 (48,204 vs. 44,534), highlighting the significant burden that this public health crisis places upon society at large [1].

Homicide rates have drawn national attention, often to the exclusion of overall gun violence [2-5]. It is often implied that the one (homicides) is reflective of the other (gun violence). We hypothesized that using homicide rates as a proxy for gun violence in general would, instead, potentially underrepresent the true extent of gun-related crimes in communities. To test our hypothesis, we chose a large urban city in the southern United States for our analysis. The objective of this study is to evaluate whether the overall trend in homicide rates serves as a good proxy for the overall trend in gun-related violent crime over time.

## Materials and methods

In this retrospective study, consecutive patients sustaining gunshot wounds within the city of Baton Rouge, Louisiana, who were treated at the trauma center from 2014 to 2020, were identified from the trauma registry following approval from the Institutional Review Board. These were then stratified by year for analysis.

Using publicly available police reports from the Baton Rouge Police Department, violent incidents that involved the use of a firearm were identified over the same period and stratified by year. Inclusion criteria for gun-related violent crime were any of the following reported incidents: attempted or committed murder, assault, battery, or drive-by shooting. All other incidents were excluded. In each incident, the use of a firearm was specified by the police report. Lastly, homicides that occurred within the city of Baton Rouge over the study period were identified and stratified by year. All police reports were accessed from the publicly available database Open Data BR (data.brla.gov), by accessing public safety information, followed by the Baton Rouge Police Crime incidents. Information includes date, statute description (e.g., first-degree murder), and attempted versus committed.

All analyses were performed using R version 4.2.2 (R Foundation for Statistical Computing, Vienna, Austria). Aggregate data from the trauma registry and police reports were combined, and a simple linear regression was performed to analyze the rate of homicides, gun-related violent crimes, and gunshot victims treated at the trauma center over the study period. Analysis of covariance (ANCOVA) was used to test whether the linear trend in rates of homicide and gun-related violent crime was significantly different. An interaction term between year and gun-related violent crime was included to evaluate differences in slopes. A p-value of <0.05 was considered statistically significant. The distribution of each variable was assessed for normality using the Shapiro-Wilk test. For variables with a normal distribution, data were presented as mean and standard deviation; for variables with a non-normal distribution, data were presented as median and interquartile range (IQR).

## Results

Over the study period (2014-2020), there were 4,928 gun-related violent crimes and 567 homicides within the city of Baton Rouge. There was no missing data for the analysis. The types of crimes varied in number (Table 1). Attempted or committed murders were the most common (n = 2,564), with a peak incidence of 530 in 2020. The second most common crime was assault (n = 1,730), followed by battery (n = 381). The least common gun-related violent crime was drive-by shooting (n = 253). The peak incidence of gunshot victims, violent gun crime, and homicides was in 2020 for each group (Table 2). There was some seasonal variation with respect to gun crime (Table 3). The months with the fewest number of gun-related violent crime incidents were January through April (all less than 400 incidents per month). The peak months were July (n = 455) and September (n = 453).

**Table 1:** Gun-related violent crimes by type as reported by the Baton Rouge Police Department from 2014 to 2020. Murder includes attempted and committed murder by firearm.

Crime Type	Number
Murder	2,564
Assault	1,730
Battery	381
Drive-by shooting	253

**Table 2:** Comparison of the total number of trauma center patients with gunshot wounds, gun-related violent crimes, and homicides committed within the city of Baton Rouge from 2014 to 2020. Trauma = Number of victims treated at the regional trauma center with gunshot wounds as the mechanism of injury occurring within the city of Baton Rouge; Firearm Crimes = Number of firearm-related violent crimes; Homicides = Number of homicides reported within the city of Baton Rouge.

Year	Trauma	Firearm Crimes	Homicides
2020	285	1,015	114
2019	205	746	76
2018	163	664	91
2017	262	855	96
2016	204	655	61
2015	149	533	70
2014	132	460	59

**Table 3:** Total number of gun-related violent crimes by month as reported by the Baton Rouge Police Department from 2014 to 2020.

Month	Total Number (Percent)
January	352 (7.1)
February	308 (6.3)
March	376 (7.6)
April	397 (8.1)
May	410 (8.3)
June	424 (8.6)
July	455 (9.2)
August	440 (8.9)
September	453 (9.2)
October	449 (9.1)
November	439 (8.9)
December	425 (8.6)

**Figure 1:**
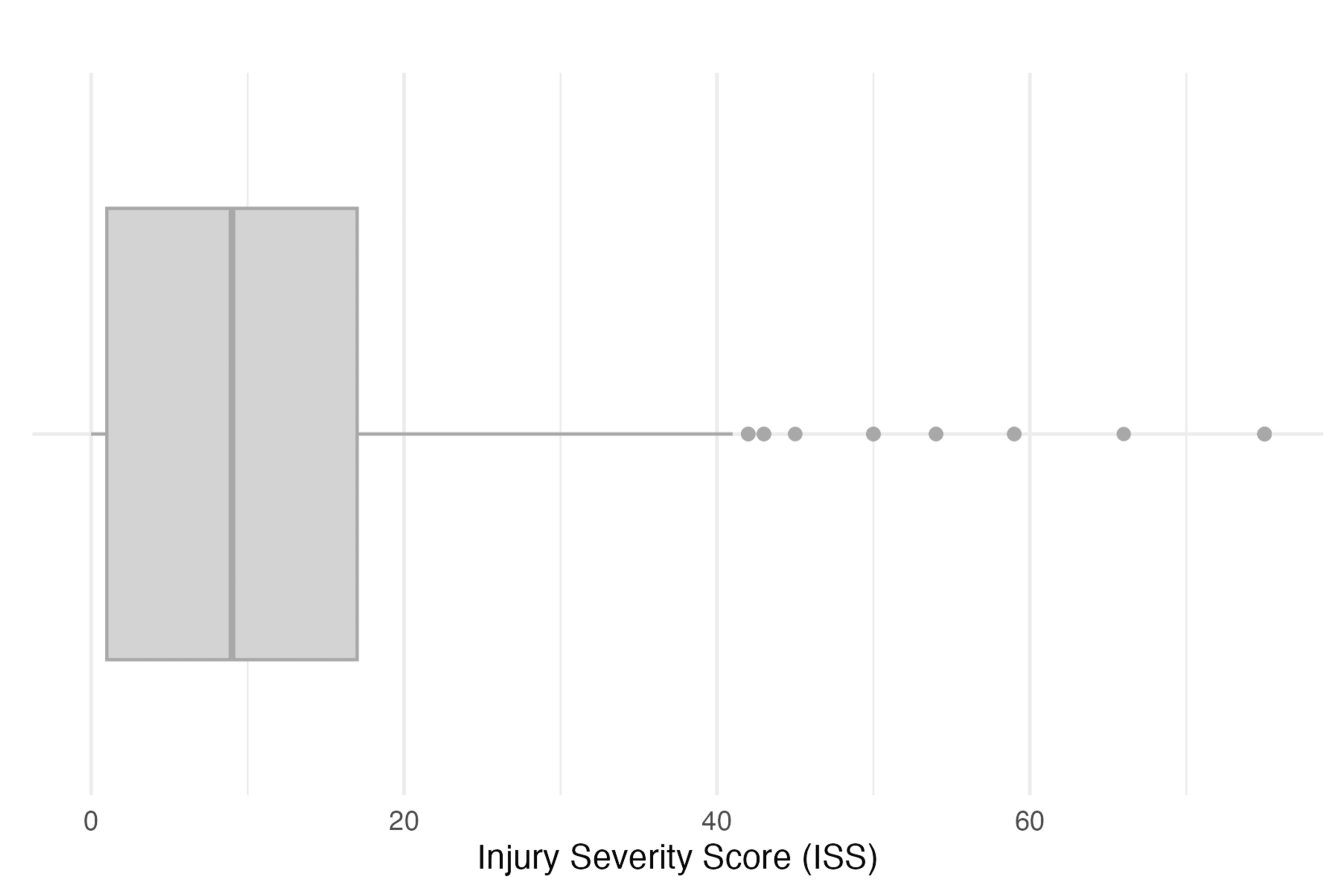
Box-and-whisker plot of Injury Severity Score. The dark vertical line represents the median, the box represents the interquartile range (IQR), the whiskers represent 1.5 × Q3, and the dots represent individual outliers.

There were 1,400 gunshot victims treated at the trauma center over the study period. Victims were generally young (median = 27, IQR = 20-36), males (n = 1,190, 85%). The median Injury Severity Score (ISS) was 9 (IQR, 1-17). ISS is used to assess the severity of traumatic injury. Major trauma is most commonly defined as an ISS > 15. A box-and-whisker plot is provided for ISS in Figure 1. The median admission heart rate was 92 (IQR, 75-109) beats per minute, while the median initial systolic blood pressure was 128 (IQR, 109-142) mmHg. There were 17 (1.2%) patients with pancreatic injuries and 83 (5.9%) with liver injuries. Only 12 (0.86%) patients sustained duodenal injuries, but 74 (5.3%) had other small bowel injuries. Specifically, 5% (n = 66) of gunshot wound victims treated at the trauma center sustained a colon injury, while less than 1% (n = 10) had a rectal injury. Approximately 10% (n = 143) of all injured patients sustained a vascular injury. There were only 12 cardiac injuries (0.86%) over the study period. The median hospital and ICU lengths of stay were three (IQR, 1-7) and zero (IQR, 0-2) days, respectively. The mortality rate over the study period was 14.3% (n = 200).

Regression analysis

**Figure 2:**
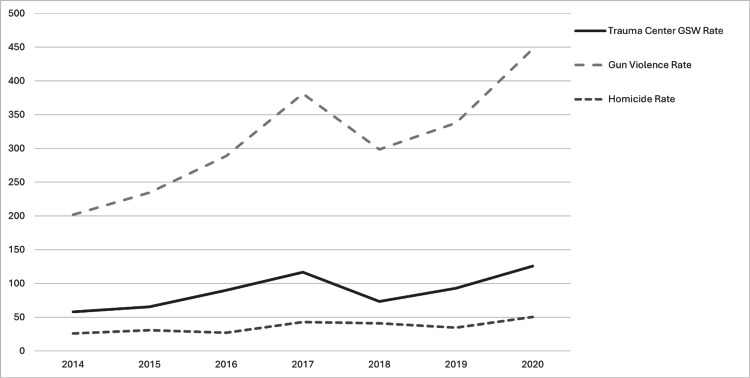
Trauma center gunshot wound victims, gun-related crimes, and homicides, per 100,000 population, from 2014 to 2020. GSW, Gunshot Wound

The incidence of gun-related violent crime (per 100,000 population) increased from 201.92 in 2014 to 447.3 in 2020 (regression coefficient = 34.014, adjusted R² = 0.71, p = 0.01) (Figure 2). Homicide incidence also increased over the study period from 25.9 to 50.24 (regression coefficient = 3.366, adjusted R² = 0.58, p = 0.028) per 100,000 population. Although the incidence of gunshot victims treated at the trauma center rose from 57.94 to 125.6 per 100,000 population (2014 and 2020, respectively), the annual trend was not statistically significant (regression coefficient = 8.601, adjusted R² = 0.44, p = 0.0624). The regression coefficient (beta) values represent the change in the incident rate per year, on average. There was a notable decrease in the rates of homicide incidence and gunshot victims treated at the trauma center between 2017 and 2018. This was similar to national data for that year [6]. It is also worth noting that there are notable trends for fluctuations in murder rate over time nationally, with an overall trend from 2014 to 2020 increasing. We are not aware of any significant changes in local or national firearm regulations, government administration, or data collection practices during this period that would explain the observed decrease. Furthermore, the standardized residuals for homicide rate, crime rate, and trauma center gunshot wound in 2017 for the dataset were 1.26, 1.63, and 1.58, respectively, which is within the typical range of residuals observed in the data (i.e., less than ±2), suggesting that the decrease noted in 2017 reflects natural variability. The Cook’s distances for homicide rate, crime rate, and trauma center gunshot wound in 2017 were 0.13, 0.22, and 0.21, respectively. These values fall well below conventional thresholds for high influence (e.g., 1.0 or 4/n), indicating that 2017 did not exert undue influence on the overall regression models, although 2017 visually stood out due to a temporary spike.

Analysis of covariance (ANCOVA)

The incidence of firearm-related violent crimes (67.34%) was compared to homicides (32.91%) over the study period. ANCOVA revealed that the interaction between year and gun-related violent crime was statistically significant (p = 0.005), indicating a different rate of increase compared to homicides.

## Discussion

The nature of firearm-related mortality has undergone extensive analysis by other authors. For example, using data from the Federal Bureau of Investigation, Manley et al. evaluated 37 years of national homicide data, identifying that most incidents involved the use of handguns (76%) and a single victim (96%) [7]. Huang et al. later evaluated gun-related deaths in the United States over a 17-year period and found that most deaths in urban areas were homicides, not suicides [8]. Gun violence in general, however, has drawn less attention and analysis than gun-related homicides and deaths, despite the heavy burden - both personal and societal - of non-fatal gunshot wounds.

This study offers a window into how homicide rates do not adequately reflect the true extent of gun-related violence in a large, urban southern city. Although the incidence of both gun-related violent crime and homicides increased from 2014 to 2020, the rate of increase of firearm-related violent crimes was greater than that of homicides. This raises the question of why gun violence overall grew faster than homicides in Baton Rouge, Louisiana.

It is possible that the shootings themselves (e.g., location and number of injuries) were less lethal. The evidence to support this hypothesis is mixed. Manley et al. found that the use of large-caliber and high-velocity firearms has increased over the past several decades, likely leading to greater lethality [9]. In a separate study, the same group found that multicompartmental injuries had also increased over time, although, paradoxically, mortality decreased [10]. Sakran et al. found that the percentage of homicides in Maryland where more than 10 gunshots were fired increased from 2005 to 2017 [11]. Brantingham et al. found that gunshot victims injured in Los Angeles, California, from 2005 to 2021 were more likely to have sustained fatal wounds over time [12]. In the current study, 1,400 patients were treated for gunshot wounds in the trauma center over a seven-year period, with a mortality rate of 14.3%. The median hospital length of stay was three days, while the median ICU length of stay was zero days. Relatively few patients sustained injuries to organs that are more often associated with a higher mortality rate, such as the heart. Taken together, data from the trauma center in the current study suggest relatively low lethality of gunshot wounds. While mortality for gunshot wound victims may or may not have increased over time, there is consensus that the wounds themselves have become more numerous and have been the result of more powerful weapons - thus failing to explain the relative increase of gun violence over homicides in Baton Rouge.

A more likely explanation is that, as the trauma system matured (the trauma center was verified in 2013, with 2014 being the first full year after verification), patient care improved, leading to relatively fewer homicides. In other words, although homicides increased over the study period, they might have increased further without a developed trauma system and its associated resources. What has been previously established is that the presence of designated trauma centers, along with the development of regional trauma systems, does improve patient outcomes. Mlaver et al. found, for example, that American College of Surgeons (ACS) verification of Level I and II centers led to improved patient outcomes, as measured by the Trauma Quality Improvement Program [13]. Similarly, Piontek et al. found that Level II ACS verification led to improved mortality and decreased hospital length of stay [14]. Furthermore, the establishment of regional trauma systems and their associated triage protocols has also been associated with improvements in mortality, as reported by Sampalis et al. [15].

Regardless of the cause, increases in gun-related violent crime outpaced those of homicides in Baton Rouge. It is also important to remember that significant psychological, physical, and financial burdens exist not only for victims of homicides, but also for those of non-fatal gun crime [16,17]. We argue that focusing on homicide rates, instead of gun-related violent crime in general, minimizes the true extent of gun-related violence and its cost upon society.

Limitations

This study has several inherent limitations. The primary limitation of this study is that it was retrospective. This precludes exclusion of selection bias and unevaluated differences, such as confounding variables, including unreported violent crimes, changes in reporting protocols, and temporal events (e.g., COVID-19). In addition, this allows for only associations to be made and cannot account for potential confounding differences. This study was performed in a single city and its only verified trauma center. Additionally, as only patients evaluated at the trauma center were included, gunshot victims treated at other hospitals in the region were not included in the study, potentially impacting the results. For the years 2021 and 2022, the Baton Rouge Police Department discontinued reporting whether or not committed or attempted crimes occurred with a firearm, which prohibited us from including those additional years in the analysis. There was also an assumption that the majority of reported homicides occurred with the use of a firearm. In addition, crimes outside of city limits were not included; as such, this study should be applied to other communities, especially non-urban communities, with caution. Lastly, although all crimes were committed with the use of a firearm, we were not able to connect police data directly with hospital data, and it is unknown how many firearm-related crimes resulted in a physical injury or death. Future studies collecting data on 911 call volume, demographic shifts within the area of interest over time, and changes in policy may be able to help clarify whether observed trends reflect actual changes in crime incidence or artifacts of reporting and enforcement.

## Conclusions

Annual homicide rates may not serve as an appropriate proxy for overall gun violence in a community. As such, changes in homicide incidence should be interpreted with caution, as they may not adequately reflect the true extent of gun-related violent crime in a community. Despite increased public awareness, the incidence of homicides and gun-related violent crimes increased in Baton Rouge, Louisiana, from 2014 to 2020. A broader understanding remains the first step in addressing this major public health problem.

## References

[1] (2025). Mortality data on CDC WONDER. https://wonder.cdc.gov/mcd.html.

[2] (2025). Baltimore homicides stubbornly high despite new initiatives. https://apnews.com/article/violence-homicide-baltimore-ce026bfcdb99cc7c6b7ae65a50eab4ff.

[3] (2025). Atlanta homicide total rises for third year with days left in 2022. https://www.ajc.com/news/crime/atlanta-homicide-total-rises-for-third-year-with-days-left-in-2022/AHE37ACHIBC4BFKMUDXXOILRWY/.

[4] (2025). 2023 was district’s deadliest year in more than two decades. https://www.washingtonpost.com/dc-md-va/interactive/2024/dc-crime-homicide-victims-shooting-violence/.

[5] (2025). As homicides rates decline in much of the US, Baton Rouge numbers remain stubbornly high. https://www.theadvocate.com/baton_rouge/as-homicide-rates-decline-in-much-of-the-us-baton-rouge-numbers-remain-stubbornly-high/article_8edb3c18-ced6-11ef-801b-838707488714.html.

[6] (2025). What the data says about gun deaths in the U.S. https://www.pewresearch.org/short-reads/2025/03/05/what-the-data-says-about-gun-deaths-in-the-us/.

[7] Manley NR, Fischer PE, Sharpe JP, Stranch EW, Fabian TC, Croce MA, Magnotti LJ (2020). Separating truth from alternative facts: 37 years of guns, murder, and violence across the US. J Am Coll Surg.

[8] Huang DD, Manley NR, Lewis RH (2022). Re-sighting the gun debate: defining patterns of firearm-related death to help focus prevention efforts. J Am Coll Surg.

[9] Manley NR, Croce MA, Fischer PE (2019). Evolution of firearm violence over 20 years: integrating law enforcement and clinical data. J Am Coll Surg.

[10] Manley NR, Fabian TC, Sharpe JP, Magnotti LJ, Croce MA (2018). Good news, bad news: an analysis of 11,294 gunshot wounds (GSWs) over two decades in a single center. J Trauma Acute Care Surg.

[11] Sakran JV, Lunardi N, Mehta A (2024). Increasing injury intensity among 6,500 violent deaths in the state of Maryland. J Am Coll Surg.

[12] Brantingham PJ, Quintana-Navarrete M, Iliff C, Uchida CD, Tita GE (2024). Situational and victim correlates of increased case fatality rates in Los Angeles shootings, 2005-2021. J Urban Health.

[13] Mlaver E, Atkins EV, Medeiros RS (2025). Impact of American College of Surgeons trauma verification on statewide collaborative outcomes. J Trauma Acute Care Surg.

[14] Piontek FA, Coscia R, Marselle CS, Korn RL, Zarling EJ (2003). Impact of American College of Surgeons verification on trauma outcomes. J Trauma.

[15] Sampalis JS, Denis R, Lavoie A (1999). Trauma care regionalization: a process-outcome evaluation. J Trauma.

[16] Wolf JM, Bouftas F, Landy DC, Strelzow JA (2024). Gunshot trauma patients have higher risk of PTSD compared with blunt trauma and elective populations: a retrospective comparative study of outpatient orthopaedic care. Clin Orthop Relat Res.

[17] Miller T, Downing J, Wheeler L, Fischer K (2024). The medical costs of firearm injuries in the United States: a systematic review. J Emerg Med.

